# Levels of DEFA1, Progranulin, and NRG4 in Patients with Autonomic Neuropathy: Potential Biomarkers for Diagnosis and Prognosis

**DOI:** 10.3390/metabo15030169

**Published:** 2025-03-02

**Authors:** Diana Nikolova, Zdravko Kamenov, Julieta Hristova, Antoaneta Trifonova Gateva

**Affiliations:** 1 Department of Internal Medicine, Aleksandrovska University Hospital, Medical University of Sofia, 1431 Sofia, Bulgaria; zkamenov@medfac.mu-sofia.bg (Z.K.); agateva@medfac.mu-sofia.bg (A.T.G.); 2 Department of Clinical Laboratory, Aleksandrovska University Hospital, Medical University of Sofia, 1431 Sofia, Bulgaria; jhristova@medfac.mu-sofia.bg

**Keywords:** diabetic autonomic neuropathy, DEFA1, progranulin, NRG4, biomarkers, inflammation, metabolic disorders

## Abstract

Background: Diabetic autonomic neuropathy (DAN) is a severe complication of diabetes that affects the autonomic nervous system, impacting cardiovascular, gastrointestinal, genitourinary, and other systems. This study examines the levels of three potential biomarkers—DEFA1, progranulin, and NRG4—to assess their diagnostic and prognostic value in DAN patients. Methods: This observational, single-center study included 80 patients with type 2 diabetes. Clinical data and laboratory results were collected, and serum levels of DEFA1, progranulin, and NRG4 were measured using ELISA. The presence of DAN was assessed using Ewing’s tests. Statistical analyses included *t*-tests, Pearson’s correlations, and ROC analysis to explore associations and the predictive values of the biomarkers. Results: Progranulin levels were significantly elevated in patients with DAN compared to those without (*p* < 0.05), showing a positive correlation with diabetes duration (r = 0.375; *p* = 0.01) and a significant predictive value for DAN (AUC = 0.666; *p* = 0.013). DEFA1 and NRG4 levels did not differ significantly between the groups. Progranulin was also higher in patients who were treated with sulfonylureas and GLP-1 receptor agonists and in those with coronary artery disease. Conclusions: Progranulin emerges as a potential biomarker for the presence and severity of DAN, correlating with disease duration and autonomic dysfunction. While DEFA1 and NRG4 showed no significant association, the findings underscore the importance of further exploring the inflammatory pathways in DAN. Progranulin measurement could enhance early diagnosis and personalized management of autonomic neuropathy in diabetes.

## 1. Introduction

Autonomic neuropathy constitutes a multifaceted syndrome that is characterized by impairment of the autonomic nervous system (ANS), leading to a broad spectrum of clinical manifestations. These include dysregulation of cardiovascular homeostasis, gastrointestinal motility, and responses to environmental stimuli. Frequently, autonomic neuropathy is concomitant with systemic pathologies such as diabetes mellitus, autoimmune disorders, and neurodegenerative diseases, highlighting its multifactorial and complex etiology [1].

Diabetic neuropathy is the most common chronic complication of diabetes mellitus. It is defined as “the presence of symptoms and/or signs of dysfunction of peripheral nerves in individuals with diabetes, after excluding other causes” [2]. Early diagnosis and treatment are of the utmost importance for several reasons. First and foremost, diabetic polyneuropathy (DPN) is diagnosed after other possible conditions have been excluded. Furthermore, there are various treatment approaches for the symptomatic form of the disease. The asymptomatic course of the disease, which can occur in up to 50% of cases, predisposes patients to injury due to a loss of sensation. Last but not least, early recognition of autonomic neuropathy can significantly improve the quality of life for patients with the condition [3].

Studies have revealed that diabetic polyneuropathy has a complex and multifactorial pathogenesis that varies depending on the type of diabetes. In type 2 diabetes, key mechanisms for the development of DPN include oxidative stress, as well as vascular and metabolic disturbances. In contrast, in type 1 diabetes, hyperglycemia plays a leading role, triggering a series of damaging processes. Among the main risk factors for DPN are high blood glucose levels, hypertension, dyslipidemia, and obesity [4]. Despite significant advances in the study of the pathophysiology of diabetic polyneuropathy, the process remains incompletely understood [5].

Diabetic autonomic neuropathy (DAN) is a serious and common complication of diabetes (more common in patients with type 1 diabetes), which often remains asymptomatic in its early stages. The subclinical form of DAN may develop within one year in type 2 diabetes and up to two years in type 1 diabetes [6,7]. The fibers of the autonomic nervous system (vasomotor, visceromotor, and sensory) innervate all organs. Therefore, DAN presents with a variety of clinical manifestations, including orthostatic hypotension, resting tachycardia, unrecognized hypoglycemia, gastroparesis, erectile dysfunction, neurogenic bladder, and sudomotor dysfunction [3]. Hypotheses regarding the pathogenic mechanisms are also diverse. They include activation of the polyol pathway with sorbitol accumulation, activation of protein kinase C, oxidative stress, and endothelial dysfunction [1].

Cardiac autonomic neuropathy (CAN) is the most common form of diabetic autonomic neuropathy (DAN), characterized by life-threatening complications such as arrhythmias, silent myocardial ischemia, and sudden death. It is also associated with other microangiopathic complications, including nephropathy, retinopathy, and peripheral neuropathy [6]. The American Diabetes Association (ADA) defines CAN as a disorder of cardiovascular autonomic control in patients with diabetes, after excluding other causes [8]. The main risk factors for the development of CAN in patients with type 2 diabetes include age, sex, ethnicity, the presence of microvascular complications (nephropathy, retinopathy, and peripheral neuropathy) [9], and insulin resistance [10]. Cardiac autonomic neuropathy is an independent predictor of morbidity and mortality from cardiovascular diseases (CVDs) in patients with diabetes [11]. In diabetic neuropathy, the parasympathetic fibers are affected first, leading to increased sympathetic tone. Later, sympathetic denervation progresses from the apex of the heart, impairing ventricular function and causing cardiomyopathy [6].

The gold standard in the diagnosis of cardiac autonomic neuropathy is cardiovascular reflex testing. Parasympathetic function is assessed through heart rate variability during deep breathing (the most specific test), the Valsalva maneuver, and the orthostatic test. Sympathetic function is evaluated by measuring changes in arterial pressure during the Valsalva maneuver and the orthostatic test. Possible or early CAN is defined when one abnormal test result is present, while two abnormal tests make the diagnosis definitive or confirmed. Severe or advanced CAN is considered when orthostatic hypotension is combined with abnormal heart rate variability tests [12].

Another manifestation of DAN is related to the gastrointestinal tract (GI). Gastrointestinal problems associated with diabetes result from changes in the nervous system of the intestines. These changes include a decrease in the neurons that inhibit intestinal activity, an increase in stimulating ones, and a reduction in the levels of certain neuropeptides. As a result, conditions such as delayed gastric emptying (gastroparesis), constipation, esophageal motility issues, diarrhea, and others can occur [6,13]. Acute disturbances in blood glucose levels can arise due to impaired glucose absorption [14].

DAN causes problems with the urogenital system by damaging the parasympathetic nerves. In the early stages, symptoms such as nocturia, difficulty urinating, urinary retention, bladder atony, incomplete bladder emptying, and urinary incontinence are common. As the damage progresses, other nerves are affected, further impairing control over the bladder. This condition increases the risk of frequent urinary tract infections and may be a sign of potential development of kidney failure in the long term [6,15].

Sexual dysfunction can also be one of the manifestations of diabetic autonomic neuropathy. In men, it presents as erectile dysfunction [16] and retrograde ejaculation [6], while in women, it is characterized by reduced sexual desire, pain during intercourse, and decreased lubrication [17].

DAN also affects the autonomic pupillomotor function and the function of sweat glands. The loss of nerve signals leads to dry skin, which increases the risk of developing characteristic diabetic foot ulcers [18]. ADA recommends the early use of sudomotor function tests in the diagnosis of autonomic neuropathy [19].

Progranulin (PGRN), also known as granulin-epithelin precursor (GEP), proepithelin, acrogranin, and GP88, is a growth factor composed of 593 amino acids and is abundantly expressed in a wide range of tissues and cell types. PGRN is involved in various cellular processes, including embryogenesis, tumorigenesis, inflammation, wound healing, neurodegeneration, and lysosomal function [20]. The secreted full-length form of progranulin has anti-inflammatory effects, while the proteolytically cleaved subunits from elastase, called granulin peptides (GRNs), have potent pro-inflammatory effects. It is encoded by the PRGN gene and is expressed by various cell types, including epithelial cells, macrophages, neurons, and adipocytes [21].

Defensins are small antimicrobial peptides of the innate immune system that serve as danger signals (“alarmins”) and actively participate in inflammatory and immune processes. They are divided into two main groups: α- and β-defensins. In humans, α-defensins include human neutrophil peptides 1-4 (HNP1-4) and intestinal defensins (HD-5 and HD-6). In addition to their antimicrobial effects, alpha-defensins have chemotactic, antimicrobial, and pro-inflammatory effects. They likely play a role in the pathogenesis of atherosclerosis by promoting the binding of LDL to the endothelium [22]. Together with other factors, HNP1–3s contribute to the enhancement of platelet activation and have a negative impact on endothelial function. Furthermore, they inhibit fibrinolysis mediated by the tissue plasminogen activator [23]. Neutrophil granulocytes are considered the main source of α-defensins in humans [22]. The expression of defensins varies between individuals, likely due to genetic differences in the genes that encode them. These genes are localized on chromosome 8 at the 8p22-p23 region [24].

Neuregulins (NRG) are growth factors that play various roles in the body and are associated with epidermal growth factors. They transmit signals through receptor tyrosine kinases from the ErbB family [25]. NRG molecules, which bind to receptor tyrosine kinases from the ErbB family, play a crucial role in the development of the nervous system, as well as in the formation of organs such as the heart and breast. They are also associated with various diseases, including different types of cancer [26]. Reduced levels of neuregulin 4 (NRG4) have been associated with increased carotid intima thickness, greater severity of coronary artery disease, and an increased risk of acute coronary syndrome. These associations emphasize the importance of NRG4 in cardiovascular health and its potential role as a biomarker for assessing cardiovascular risk [27]. Neuregulins are significantly expressed in the nervous system and play a key role in the development and functioning of neurons and glia. They are involved in regulating neuronal network formation, myelination, neurotransmission, and synaptic plasticity. These processes are essential for the normal functioning and development of the nervous system [28]. Neuregulin 4 plays an important role in the development of sympathetic nerve fibers and the regulation of sympathetic innervation of adipose tissue. It stimulates the growth of developing sympathetic axons and is crucial for the proper formation of the nerve networks that innervate adipose tissue [29].

This study aims to analyze the levels of defensin alfa 1 (DEFA1), progranulin, and NRG4 in patients with diabetic autonomic neuropathy. These molecules have the potential to reflect clinically significant changes. The study of them could reveal new biomarkers for diabetic autonomic neuropathy, which might be useful for assessing the pathogenesis of the condition, as well as for diagnosis and monitoring of the disease. This research will provide a better understanding of the relationship between these molecules and diabetic neuropathy, offering valuable insights for early diagnostic approaches and new therapeutic targets for preventing or treating DAN.

## 2. Materials and Methods

### 2.1. Study Design

This study was observational, monocentric, and included 80 patients with type 2 diabetes. The goal was to investigate the levels of DEFA1, progranulin, and NRG4 and their relationship with autonomic neuropathy. Data collection was conducted over a one-year period.

The protocol of the study was in accordance with the declaration of Helsinki and was approved by the Ethics Committee of the Medical University Sofia (Protocol № 11/11. 07. 2023). All participating subjects signed a written informed consent form.

### 2.2. Inclusion and Exclusion Criteria

Inclusion Criteria:

- Age > 18 and <65 years;
- Type 2 diabetes mellitus with a duration of 5–15 years;
- Glycated hemoglobin (HbA1c) between 6% and 8% for patients with diabetes;
- Presence of somatic neuropathy;
- Presence of autonomic neuropathy;
- Signed informed consent.

Exclusion Criteria:

- Secondary causes of polyneuropathy, including alcoholism, vitamin B deficiency, occupational diseases, Guillain–Barré syndrome, infectious diseases, and heavy metal intoxication;
- Acute complications of diabetes, such as diabetic ketoacidosis, diabetic hyperosmolar coma, or hypoglycemic coma;
- Type 1 diabetes mellitus;
- Confirmed neoplasia;
- Chronic kidney disease (CKD), stages III–IV;
- Heart failure (HF), class III–IV according to NYHA classification.

### 2.3. Diagnosis of Autonomic Neuropathy

Autonomic neuropathy was assessed using Ewing’s tests.

### 2.4. Laboratory Investigations

The serum levels of DEFA1, progranulin, and NRG4 were measured using standard ELISA techniques.

### 2.5. Statistical Analysis

The data were analyzed using the SPSS statistical software (version 23). Continuous variables are presented as mean ± standard deviation. Statistical differences between groups with and without autonomic neuropathy were assessed using the *t*-test. Correlations were calculated using Pearson’s correlation coefficient. ROC analysis was used to assess the predictive value of progranulin.

## 3. Results

### 3.1. Participants

The average age of the participants was 59.9 ± 6.2 years, with 48.8% being male. The patients had a body mass index (BMI) of 36.2 ± 6.2 kg/m^2^ and a diabetes duration of 9.1 ± 5.4 years. Obesity was observed in 88.8% of the participants.

The main characteristics of the patients are presented in Table 1.

### 3.2. Treatment

#### Treatment and Glycemic Control

Of the patients studied, only 1 (1.3%) was not undergoing treatment, while 26 (32.5%) were on treatment with one anti-diabetic drug, 25 (31.1%) with two, 16 (20%) with three, 10 (12.5%) with four, and 2 (2.5%) with five anti-diabetic medications. The most commonly used drugs included metformin (85%), sulfonylureas (41%), SGLT-2 inhibitors (28.7%), GLP-1 receptor analogs (22.8%), insulin (25.3%), and DPP-4 inhibitors (15.2%). The average HbA1c was 7.7 ± 1.3%, with 15% of patients having HbA1c < 6.5%, 23.4% with HbA1c between 5.6% and 7.5%, 37% with HbA1c between 7.6% and 8.5%, and 23.4% with HbA1c > 8.5%.

### 3.3. Diabetes Complications

Among the microvascular diabetic complications, diabetic nephropathy was present in 23 individuals (28.7%), diabetic retinopathy in 12 (15%), and peripheral diabetic neuropathy in 34 (43%).

Autonomic neuropathy was found in 50 participants (62.5%). No significant differences were found in the mean age, anthropometric measures, duration of diabetes, and HbA1c levels between patients with and without autonomic neuropathy, although there was a trend toward a higher mean age and longer duration of diabetes in those with autonomic neuropathy (Table 2).

### 3.4. Levels of Progranulin, DEFA1, and NRG4

Of the biomarkers studied, only progranulin was significantly elevated in patients with autonomic neuropathy (Table 3). No significant differences were found in the levels of DEFA1, progranulin, and NRG4 between patients with and without other microvascular complications (peripheral neuropathy, retinopathy, and nephropathy).

**Figure 1:**
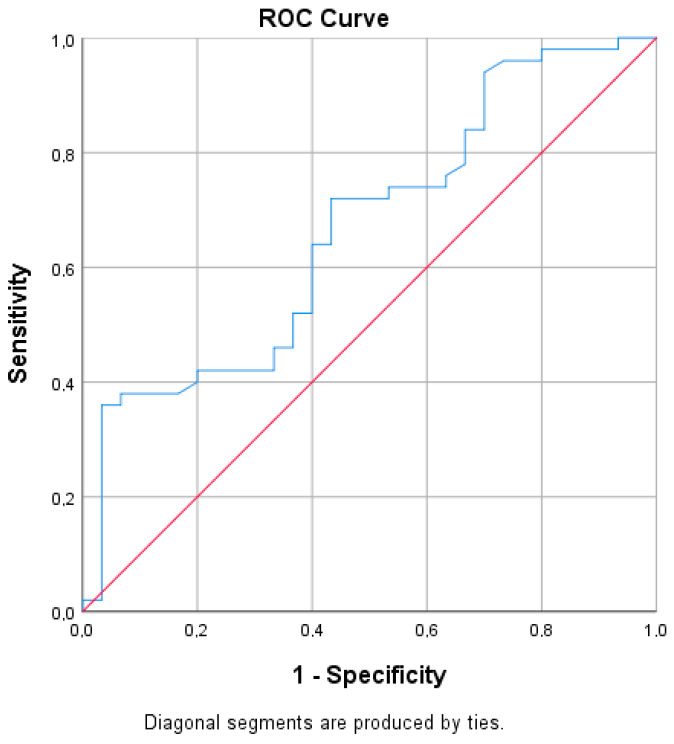
ROC curve for progranulin.

**Figure 2:**
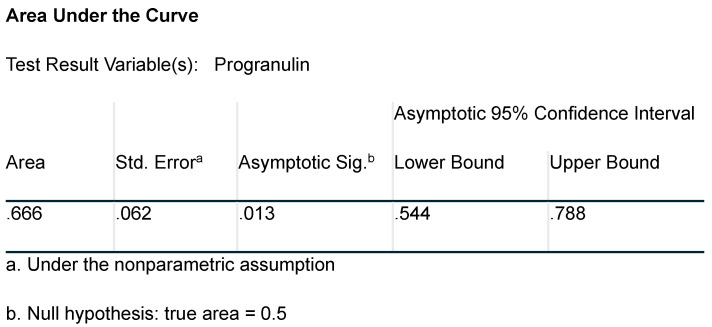
Area Under the Curve (AUC) analysis for progranulin.

The serum progranulin levels showed a very good predictive value for the presence of autonomic neuropathy with an AUC of 0.666, and *p* = 0.013 (Figure 1 and Figure 2).

Higher levels of progranulin were found in patients undergoing treatment with insulin secretagogues, such as sulfonylureas (35.1 ± 14.3 vs. 29.4 ± 6.9; *p* = 0.021), and GLP-1 receptor analogs (36.1 ± 17.9 vs. 30.3 ± 7.3; *p* = 0.042), regardless of other accompanying therapy.

## 4. Discussion

**Diabetes mellitus** is a complex, chronic condition that requires continuous medical care and multifactorial strategies to reduce risks beyond glycemic control [30]. The global diabetes pandemic is growing at an alarming rate, with the incidence of all types of diabetes doubling from 4.7% in 1980 to 8.5% in 2014. It is projected that by 2045, 700 million people worldwide will have diabetes, an increase of one-third from the current number [31]. Chronic microvascular (retinopathy, neuropathy, and nephropathy) and macrovascular (myocardial infarction, cerebrovascular diseases, and peripheral artery disease) complications remain a significant challenge [32]. In 65% of patients with type 2 diabetes, the disease can be complicated by diabetic autonomic neuropathy [6]. Hyperglycemia activates metabolic pathways such as the polyol, hexosamine, and protein kinase C pathways, leading to the accumulation of advanced glycation end-products (AGEs), axonal dysfunction, and neurovascular damage. The disturbances include the accumulation of sorbitol, a decrease in Na-K-ATPase activity, and endoneural hypoxia. The result is neurovascular dysfunction, apoptosis, and sensory deficits, exacerbated by the activation of redox-sensitive factors such as the nuclear transcription factor κB (NFκB) and reactive oxygen species (ROS) [33]. Increased glucose leads to mitochondrial overproduction of reactive oxygen and nitrogen species (RONS), causing tissue damage. RONS damage DNA, activating the enzyme poly-ADP ribose polymerase (PARP), which consumes NAD and reduces the activity of GAPDH. This results in endothelial dysfunction and the activation of apoptosis, including the formation of AGEs. AGEs alter the protein structure and interact with receptors (RAGEs), which enhance inflammation and oxidative stress [6]. Under conditions of hyperglycemia, aldose reductase converts glucose into sorbitol, which is subsequently oxidized to fructose. The accumulation of sorbitol leads to oxidative stress, while the negative effects of fructose manifest in enzyme glycation and the formation of advanced glycation end-products [34]. It is well known that changes in the polyol pathway lead to alterations in nerve impulse conduction [1].

The state of chronic inflammation is associated with the pathogenesis of insulin resistance, type 2 diabetes, as well as its chronic complications [35]. The connection between well-known inflammatory markers such as C-reactive protein (CRP), IL-6, IL-8, TNF-α, and endothelin-1 and DAN has been established in patients with type 1 and type 2 diabetes [6].

This study provides new data on the relationship between the levels of DEFA1, progranulin, and NRG4 and the presence of autonomic neuropathy in patients with type 2 diabetes. The main finding is a significant increase in progranulin levels in patients with autonomic neuropathy, highlighting the potential role of this biomarker in the pathogenesis and diagnosis of the condition.

### 4.1. Progranulin and Autonomic Neuropathy

Progranulin plays a crucial role in modulating the inflammatory response, as well as in cell proliferation and regeneration processes. The increase in its levels during inflammatory conditions suggests its involvement in chronic subclinical inflammation, which is associated with the pathogenesis of diabetic microangiopathy. Higher levels have been measured in patients with type 2 diabetes and microvascular complications compared to patients with type 2 diabetes without complications. The serum levels of progranulin increase proportionally with the severity of diabetic nephropathy and diabetic retinopathy [36]. It is possible that progranulin participates in the pathogenesis of diabetic microangiopathy by expressing interleukin-6 in adipocytes. Studies by Youn et al. have found a correlation between CRP levels and progranulin in patients with type 2 diabetes and obesity, making it a potential marker for chronic inflammation [37]. However, the exact mechanisms underlying the increase in progranulin levels in patients with diabetic microangiopathy remain incompletely explored. Data in the literature on the link between progranulin and diabetic autonomic neuropathy are limited. Our data align with studies by Albeltagy et al., which demonstrate elevated levels of progranulin in diabetic nephropathy and vascular dysfunction. The inflammatory mechanism associated with progranulin is likely a key factor in the damage to the autonomic nervous system. This may be explained by the chronic inflammation and oxidative stress that are characteristic of type 2 diabetes [36]. PGRN binds to tumor necrosis factor alfa (TNF-α) through its receptors (TNFR1 and TNFR2), thereby exerting an anti-inflammatory effect in a variety of diseases, including Alzheimer’s disease, rheumatoid arthritis, systemic lupus erythematosus, inflammatory bowel disease, bacterial pneumonia, and atherosclerosis [38,39]. Progranulin binds to the TNFR1 receptor with the same affinity as TNF-α, but its affinity for the TNFR2 receptor is nearly 600 times higher than that of TNF-α [39]. PGRN reduces inflammation by inhibiting the release of interleukin-6 (IL-6) and chemoattractant protein-1 from macrophages, while stimulating the production of the anti-inflammatory interleukin-10 (IL-10). In the absence of PGRN, macrophages intensify the inflammatory response by increasing the secretion of cytokines. The anti-inflammatory action of PGRN is supported by binding proteins such as the secretory leukocyte protease inhibitor and Apo A-1 [40]. This aligns with our findings that elevated progranulin levels are associated with the pathogenesis of diabetic neuropathy, potentially via modulation of chronic inflammation.

### 4.2. DEFA1 and NRG4

Németh et al. analyzed the serum levels of α-defensin in type 2 diabetes and reported a significant increase compared to controls, as well as finding that the serum levels of α-defensin in patients with diabetic neuropathy were significantly higher than in patients with diabetes without complications. The highest concentrations of HNP 1-3 were found in patients with diabetes and nephropathy [22]. Another study among patients with type 2 diabetes demonstrated significant positive correlations between α-defensin and the levels of AGEs, fasting plasma glucose, BMI, and diabetes duration. As previously mentioned, advanced glycation end-products play a key role in the pathogenesis of diabetic complications. Elevated blood glucose stimulates the formation of AGEs, which activate the RAGE receptors. This leads to the activation of NFkB in monocytes and macrophages, enhancing inflammation in diabetes and potentially increasing α-defensin levels in the blood [35].

Our results contrast with data from Nemeth et al., where the highest concentrations of HNP 1-3 (part of the α-defensins) were found in patients with diabetes and nephropathy. While this could be due to reduced renal degradation of peptides in patients with advanced nephropathy, the relationship with diabetic neuropathy remains not fully clarified [22].

Neuregulin 4 (Nrg4) is a novel neurotrophic adipokine with a transmembrane structure and a short extracellular part containing an epidermal growth factor (EGF)-like domain. This domain is surrounded by a proteolytic cleavage site, which, when cleaved, releases the EGF domain. This domain then specifically binds to ErbB4 receptors [41]. It is predominantly secreted by brown adipose tissue. Nrg4 plays an important role in metabolic processes and is associated with various diseases, including diabetic polyneuropathy. Reduced levels of Nrg4 are linked to obesity, insulin resistance, diabetes, dyslipidemia, metabolic syndrome, non-alcoholic fatty liver disease, inflammation, oxidative stress, and macrovascular diseases such as coronary artery disease and myocardial infarction. These factors contribute to the development of diabetic polyneuropathy, suggesting that Nrg4 plays a critical role in its development. Lower serum levels of Nrg4 may be associated with peripheral neuropathy in patients with newly diagnosed type 2 diabetes. These findings highlight the potential role of Nrg4 as a therapeutic target for preventing or treating diabetic polyneuropathy [42].

Various studies have identified NRG4 as a likely marker for diagnosing diabetes and metabolic syndrome, demonstrating decreased levels of this adipokine in these conditions [43].

Despite the known roles of DEFA1 and NRG4 in inflammatory and metabolic processes, this study did not find significant differences in the levels of these biomarkers between patients with and without autonomic neuropathy.

### 4.3. Clinical Significance

The prognostic value of progranulin suggests its potential application as a diagnostic tool for autonomic neuropathy. Additional observations of elevated levels in patients using sulfonylureas and GLP-1 receptor analogs also suggest a potential link between therapy and inflammatory status. These results open avenues for future interventional studies to evaluate the impact of specific therapies on progranulin levels.

### 4.4. Limitations of the Study

The limitations include the small sample size and the absence of additional mechanistic studies. The lack of long-term follow-up also limits the ability to establish causal relationships. Future studies with larger patient cohorts and a prospective design are necessary to confirm these results.

## 5. Conclusions

This study identifies progranulin as a potential biomarker for autonomic neuropathy in type 2 diabetes. The inflammatory role of progranulin, along with its correlation with diabetes duration, highlights its importance as a diagnostic and prognostic tool. Future research may contribute to a better understanding of the pathogenesis of autonomic neuropathy and the role of inflammatory biomarkers in its development.

## Figures and Tables

**Table 1:** The demographic, clinical, and laboratory profiles of all the patients.

Age (years)	59.9 ± 6.2
Weight (kg)	100.8 ± 19.1
BMI (kg/m^2^)	36.2 ± 6.2
Obesity (%)	88.8%
Duration of diabetes (years)	9.1 ± 5.4

**Table 2:** The main characteristics of the patients without and with autonomic neuropathy.

	Without Autonomic Neuropathy	With Autonomic Neuropathy
Age (years)	59.5 ± 6.6	60.2 ± 6.0
Weight (kg)	104.0 ± 19.1	98.9 ± 19.0
BMI (kg/m^2^)	34.9 ± 4.9	37.0 ± 6.8
Obesity (%)	83.3%	90%
Duration of diabetes (years)	8.3 ± 4.1	9.7 ± 6.1
HbA1c (%)	7.9 ± 1.4	7.6 ± 1.2

**Table 3:** Levels of progranulin, DEFA1, and NRG4 in patients with autonomic neuropathy.

	Without Autonomic Neuropathy	With Autonomic Neuropathy
DEFA1 ng/mL	1.3 ± 0.79	1.1 ± 0.66
Progranulin ng/mL	27.7 (17.2–38.1)	30.9 (17.3–44.5) *
NRG4 pg/mL	235.6 (32.7–438.5)	246.5 (70.8–421.3)

The data are presented as mean ± SD or median (Q1–Q3). * *p* < 0.05

## Data Availability

Data unavailable due to privacy or ethical restrictions.

## Abbreviations

The following abbreviations are used in this manuscript:

DAN	Diabetic autonomic neuropathy
ANS	Autonomic nervous system
DPN	Diabetic polyneuropathy
CAN	Cardiac autonomic neuropathy
ADA	American Diabetes Association
CVDs	Cardiovascular diseases
GI	Gastrointestinal tract
PGRN	Progranulin
GEP	Granulin-epithelin precursor
GRNs	Granulin peptides
HNPs 1-4	Human neutrophil peptides 1-4
LDLs	Low-density lipoproteins
NRGs	Neuregulins
NRG4	Neuregulin 4
DEFA1	Defensin Alpha 1
HbA1c	Glycated hemoglobin
CKD	Chronic kidney disease
HF	Heart failure
NYHA	New York Heart Association
ELISA	Enzyme-linked immunosorbent assay
ROC	Receiver operating characteristic
BMI	Body mass index
SGLT-2	Sodium-glucose transport protein 2
GLP-1	Glucagon-like peptide-1
DPP-4	Dipeptidyl peptidase-4
AUC	Area Under the Curve
AGEs	Advanced glycation end-products
NFκB	Nuclear transcription factor κB
ROS	Reactive oxygen species
RONS	Reactive oxygen and nitrogen species
DNA	Deoxyribonucleic acid
PARP	Poly-ADP ribose polymerase
NAD	Nicotinamide Adenine Dinucleotide
GAPDH	Glyceraldehyde-3-phosphate dehydrogenase
RAGEs	Receptors for Advanced Glycation End-products
CRP	C-reactive protein
IL-6	Interleukin -6
IL-8	Interleukin-8
TNF-α	Tumor necrosis factor alfa
TNFR1	Tumor necrosis factor receptor 1
TNFR2	Tumor necrosis factor receptor 2
IL-10	Interleukin-10
